# Evidence for the Existence of Secretory Granule (Dense-Core Vesicle)-Based Inositol 1,4,5-Trisphosphate-Dependent Ca^2+^ Signaling System in Astrocytes

**DOI:** 10.1371/journal.pone.0011973

**Published:** 2010-08-05

**Authors:** Yong Suk Hur, Ki Deok Kim, Sun Ha Paek, Seung Hyun Yoo

**Affiliations:** 1 Department of Biochemistry, Inha University School of Medicine, Jung Gu, Incheon, Korea; 2 Department of Neurosurgery, Seoul National University College of Medicine, Jongno Gu, Seoul, Korea; Universidade Federal do Rio de Janeiro, Brazil

## Abstract

**Background:**

The gliotransmitters released from astrocytes are deemed to play key roles in the glial cell-neuron communication for normal function of the brain. The gliotransmitters, such as glutamate, ATP, D-serine, neuropeptide Y, are stored in vesicles of astrocytes and secreted following the inositol 1,4,5-trisphosphate (IP_3_)-induced intracellular Ca^2+^ releases. Yet studies on the identity of the IP_3_-dependent intracellular Ca^2+^ stores remain virtually unexplored.

**Principal Findings:**

We have therefore studied the potential existence of the IP_3_-sensitive intracellular Ca^2+^ stores in the cytoplasm of astrocytes using human brain tissue samples in contrast to cultured astrocytes that had primarily been used in the past. It was thus found that secretory granule marker proteins chromogranins and secretogranin II localize in the large dense core vesicles of astrocytes, thereby confirming the large dense core vesicles as bona fide secretory granules. Moreover, consistent with the major IP_3_-dependent intracellular Ca^2+^ store role of secretory granules in secretory cells, secretory granules of astrocytes also contained all three (types 1, 2, and 3) IP_3_R isoforms.

**Significance:**

Given that the secretory granule marker proteins chromogranins and secretogranin II are high-capacity, low-affinity Ca^2+^ storage proteins and chromogranins interact with the IP_3_Rs to activate the IP_3_R/Ca^2+^ channels, i.e., increase both the mean open time and the open probability of the channels, these results imply that secretory granules of astrocytes function as the IP_3_-sensitive intracellular Ca^2+^ store.

## Introduction

Astrocytes are now known to secrete a number of signaling molecules that participate in the cell-to-cell communication, involving both neurons and glial cells [1]–[7]. Of these signaling molecules, ATP, glutamate, D-serine, neuropeptide Y (NPY), called gliotransmitters, are known. These gliotransmitters are stored in secretory vesicles in astrocytes and are released in a Ca^2+^-dependent regulatory secretory pathway [8]–[16]. There exist generally two types of secretory vesicles in astrocytes, one being the translucent small synaptic-like vesicles and the other the large dense-core vesicles (LDCV) [11], [17]–[19]. Analogous to the neurotransmitters stored in synaptic vesicles of neurons, small signaling molecules of astrocytes are traditionally thought to be stored in small synaptic-like vesicles and released in a regulated fashion, which in turn participate in neuron-glial cell communication in the brain [4], [14], [20]–[27]. However, the large dense core vesicles were also shown to contain a variety of small and large molecules that are of importance in cell-to-cell communication [10], [19], [28]–[30].

Similar to other secretory cells, the regulatory secretory pathway in astrocytes is shown to depend on inositol 1,4,5-trisphosphate (IP_3_)-mediated Ca^2+^ release from intracellular Ca^2+^ stores [20], [22], [25], [29], [31]. In spite of the IP_3_-dependent intracellular Ca^2+^ release that leads to secretion of gliotransmitters, the identity of the intracellular stores that function as the IP_3_-sensitive Ca^2+^ stores has not been addressed except the traditional role of the endoplasmic reticulum (ER). However, in recent studies it has been demonstrated that the ER plays only a minor role in the IP_3_-dependent Ca^2+^ mobilization system in the cytoplasm of neuroendocrine cells [32]–[34]. Rather secretory granules were shown to be responsible for >70% of IP_3_-induced Ca^2+^ release in the cytoplasm of the cells in which they exist [32]–[34]. Secretory granules are present in virtually all secretory cells and contain by far the largest amounts of Ca^2+^ of all subcellular organelles [35]–[38]. Further, secretory granules contain the highest concentrations of cellular IP_3_R/Ca^2+^ channels in neuroendocrine cells [39], and the IP_3_R/Ca^2+^ channels of secretory granules are ∼7-fold more sensitive to IP_3_ than those of the ER [40], which means that secretory granules will release Ca^2+^ in response even to one-seventh the IP_3_ concentration that is required to induce Ca^2+^ release from the ER.

Taken together, these results clearly indicate that in secretory cells where secretory granules are intrinsically present secretory granules function as the major IP_3_-dependent intracellular Ca^2+^ store [34]. Indeed, the IP_3_-mediated Ca^2+^ release from secretory granules was shown to be sufficient to initiate exocytotic processes of insulin-secreting pancreatic β-cells in the absence of external Ca^2+^ [41]. Given the pivotal role of secretory granules in the control of IP_3_-dependent intracellular Ca^2+^ concentrations and of the regulatory secretory processes, it became of critical importance to clarify the identity of the large dense core vesicles in astrocytes. For this we first investigated the presence of typical secretory granule marker proteins, chromogranin B (CGB) and secretogranin II (SgII), in astrocytes. Moreover, in view of the highly concentrated localization of the IP_3_R/Ca^2+^ channels in secretory granules of neuroendocrine cells [39] and of the key role of Ca^2+^ release through the IP_3_R/Ca^2+^ channels in proliferation, migration, and survival of glioblastoma [42], we have also examined the potential presence of the IP_3_Rs in the large dense core vesicles of astrocytes.

In the present study, we found the localization of two typical secretory granule marker proteins chromogranin B and secretogranin II [43]–[45] in the large dense-core vesicles of astrocytes, thereby identifying the large dense-core vesicles as secretory granules [46]–[48]. We also found the presence of all three IP_3_R isoforms in secretory granules of astrocytes. Hence, in view of the roles of secretory granules in secretory cells as the major IP_3_-sensitive intracellular Ca^2+^ store, the existence of secretory granules in glial astrocytes appears to point out the presence and operation of an IP_3_-sensitive intracellular Ca^2+^ store role of secretory granules in astrocytes.

## Materials and Methods

### Antibodies

The polyclonal anti-rabbit chromogranin A (CGA), chromogranin B (CGB), secretogranin II (SgII) antibodies were raised against purified intact bovine CGA, CGB and SgII [49], [50], and affinity purified against bovine CGA, recombinant CGB and SgII [51]. The specificity of the antibodies was confirmed [50], [52]–[54]. IP_3_R peptides specific to terminal 10–13 amino acids of type 1 (HPPHMNVNPQQPA), type 2 (SNTPHENHHMPPA) and type 3 (FVDVQNCMSR) were synthesized with a carboxy-terminal cysteine and anti-rabbit polyclonal antibodies were raised. The polyclonal anti-rabbit antibodies were affinity purified on each immobilized peptide following the procedure described [55], and the specificity of each antibody has been confirmed [52]. Monoclonal anti-mouse glial fibrillary acidic protein (GFAP) antibody (clone G-A-5) was obtained from Sigma-Aldrich (St. Louis, U.S.A.).

### Human tissue samples

The human brain tissue (temporal lobe) samples examined in this study were obtained from patients undergoing surgical treatments following written consent in accordance with appropriate clinical protocols in the Department of Neurosurgery of Seoul National University Hospital. The use of samples for the present study was approved by the Institutional Review Board of Seoul National University Hospital (IRB approval number 0806-006-246).

### Immunogold electron microscopy

For the electron microscopic study of human brain tissues, the tissue samples were minced into small pieces (∼1 mm^3^) and fixed for 2 h at 4°C in PBS containing 0.1% glutaraldehyde, 4% paraformaldehyde immediately after surgical removal. After three washes in PBS, the tissues were postfixed with 1% osmium tetroxide on ice for 2 h, and washed three times in PBS. The tissues were then embedded in Epon 812 after dehydration in an ethanol series. After collection of the ultrathin (70 nm) sections on Formvar/carbon-coated nickel grids, the grids were stained with 2.5% uranyl acetate (7 min) and lead citrate (2 min). For immunogold labeling experiments, the ultrathin sections that had been collected on Formvar/carbon-coated nickel grids were floated on drops of freshly prepared 3% sodium metaperiodate for 40 min. After etching and washing, the grids were placed on 50 µl droplets of buffer A (phosphate saline solution, pH 8.2, containing 4% normal goat serum, 1% BSA, 0.1% Tween 20, 0.1% sodium azide) for 1 h. The grids were then incubated for 3 h at room temperature in a humidified chamber on 50 µl droplets of polyclonal anti-rabbit CGB or SgII antibody appropriately diluted in solution B (solution A but with 1% normal goat serum), followed by rinses in solution B. The grids were reacted with the 15-nm gold-conjugated goat anti-rabbit IgG, diluted in solution A. Controls for the specificity of CGB- or SgII-specific immunogold labeling included 1) omitting the primary antibody, 2) replacing the primary antibody with the preimmune serum, and 3) adding the primary antibody in the excess presence of purified CGB or SgII.

For double immunogold labeling, the grids were incubated for 3 h at room temperature in a humidified chamber on 50 µl droplets of monoclonal anti-mouse glial fibrillary acidic protein (GFAP) antibody appropriately diluted in solution B (solution A but with 1% normal goat serum), followed by rinses in solution B. The grids were then reacted with the 10-nm gold-conjugated goat anti-mouse IgG, diluted in solution A. After extensive washes in PBS, the grids were then incubated with polyclonal anti-rabbit either CGB or SgII antibody as described above, followed by rinses in solution B. The grids were reacted with the 15-nm gold-conjugated goat anti-rabbit IgG, diluted in solution A. After washes in PBS and deionized water, the grids were stained with uranyl acetate (7 min) and lead citrate (2 min). Following washing in deionized water and drying the samples were examined with a JEOL JEM-1011 electron microscope.

### Distribution analysis of chromogranin B, secretogranin II, and IP_3_R isoforms in astrocytes

Astrocytes are distinguished from other cells by the shapes and sizes of the cell and the nucleus. However, the presence of intermediate filaments in the cytoplasm is the exclusive hallmark of astrocytes [56], [57]. The intermediate filaments express glial fibrillary acidic protein (GFAP) and are not found in other neighboring cells [56]–[59]. Localization of CGB and SgII in secretory granules of human astrocytes was examined by analyzing the number of CGB-, and SgII-labeling gold particles localized per µm^2^ area of secretory granule and mitochondria (Table 1). However, localization of each IP_3_R isoform was examined by analyzing the number of each IP_3_R isoform-labeling gold particles per µm membrane of secretory granule and mitochondria (Table 2). Approximately 35–80 secretory granules and 30–60 mitochondria from 20–26 electron micrographs obtained from 5 different human tissue samples were used in the analysis of each group as described in the respective table.

**Table 1:** Distribution of the chromogranin B- and secretogranin II-labeling gold particles in secretory granules of human astrocytes.

	Chromogranin B^a^		Secretogranin II^b^	
	Number of gold particles/area viewed (µm^2^)	Gold particles/µm^2^	Number of gold particles/area viewed (µm^2^)	Gold particles/µm^2^
Secretory granule	64/5.17	12.38	37/3.59	10.31
Mitochondria	6/16.06	0.37	2/10.23	0.20

a 26 images from three different tissue preparations were used.

b 21 images from three different tissue preparations were used.

**Table 2:** Distribution of the IP_3_R1-, IP_3_R2 and IP_3_R3-labeling gold particles in secretory granule membranes of human astrocytes.

	IP_3_R1^a^		IP_3_R2^b^		IP_3_R3^c^	
	Number of gold particles/Length (µm)	Gold particles/µm	Number of gold particles/Length (µm)	Gold particles/µm	Number of gold particles/Length (µm)	Gold particles/µm
Secretory granule membrane	39/44.739	0.872	53/90.387	0.586	50/69.556	0.719
Mitochondrial membrane^d^	2/56.158	0.035	1/43.971	0.022	1/56.061	0.018

a 20 images from four different tissue preparations were used.

b 20 images from three different tissue preparations were used.

c 22 images from four different tissue preparations were used.

d Only the length of the outer membranes is used.

## Results

**Figure 1:**
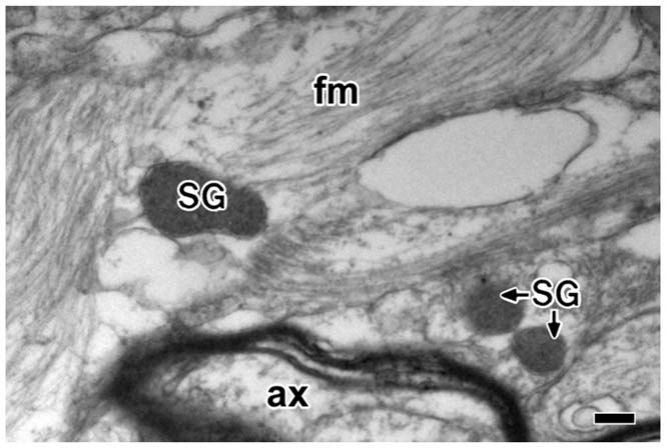
Electron micrographs showing the secretory granule-like vesicles (large dense-core vesicles) in astrocytes of brain tissues. Human brain tissues were examined by electron microscope and secretory granule-like vesicles (large dense-core vesicles) of astrocytes were shown. SG, secretory granule-like vesicles; ax, axon; fm, filament. Bar = 200 nm.

Analogous to the large dense-core vesicles of neurons glial astrocytes have also the large dense-core vesicles, yet studies on the number, location, and function of the large dense-core vesicles in astrocytes are generally lacking. In our attempt to study the LDCVs of astrocytes, we first examined the number and location of these vesicles in the cell (Fig. 1). In contrast to more abundant synaptic-like vesicles, there were fewer LDCVs and generally 0–4 LDCVs were observed in a picture image covering ∼6 µm^2^ of astrocytes (Fig. 1). It was nevertheless appeared that the cell processes were more likely to contain the large dense-core vesicles than the cell body.

**Figure 2:**
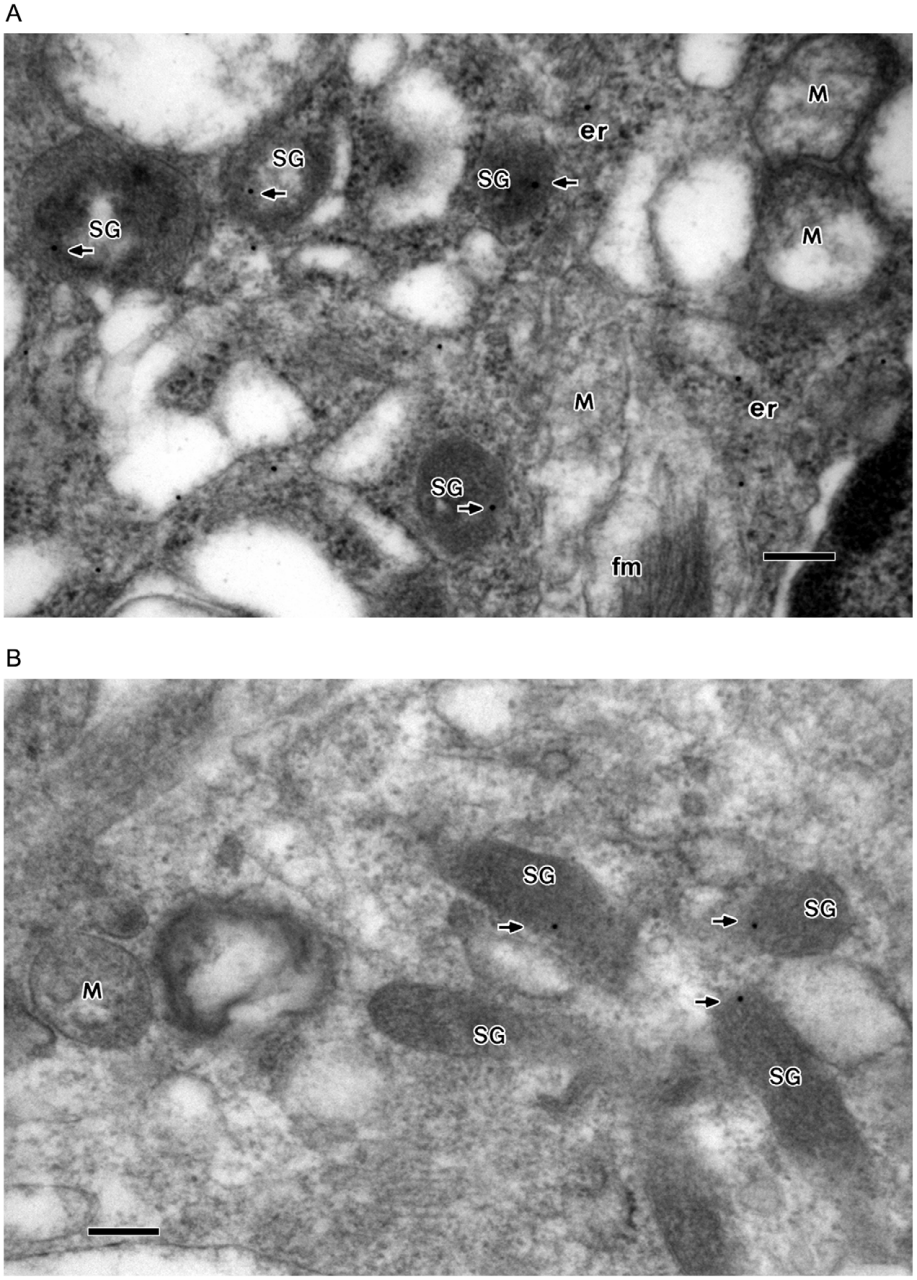
Immunogold electron microscopy showing the localization of CGB and SgII in secretory granule-like vesicles (large dense-core vesicles) in astrocytes of brain tissues. Astrocytes from human brain tissues were immunolabeled for CGB (**A**) and SgII (**B**) (15 nm gold) with the affinity purified CGB and SgII antibodies, respectively. The CGB- or SgII-labeling gold particles (indicated by arrows) were primarily localized in the secretory granule-like vesicles (SG) with some in the endoplasmic reticulum (er), but not in the mitochondria (M). In the control experiments without the primary antibodies no gold particles were seen in the secretory granule-like vesicles (not shown). Bar = 200 nm.

To determine whether these LDCVs express the secretory granule marker proteins, the expression of the two major granin proteins chromogranin B (Fig. 2A) and secretogranin II (Fig. 2B) was investigated by immunogold electron microscopy using affinity-purified CGB and SgII antibodies. As shown in Fig. 2A, chromogranin B-labeling gold particles were localized inside the large dense-core vesicles, showing the expression of CGB in the LDCVs, but they were absent in mitochondria. It was further shown that secretogranin II-labeling gold particles localize in the LDCVs as well while being absent in mitochondria (Fig. 2B), indicating the expression of SgII in the large dense-core vesicles. The expression of two typical secretory granule marker proteins CGB and SgII not only identifies the LDCVs as genuine secretory granules but also demonstrates the presence of secretory granules in glial astrocytes. Being the major residents of secretory granules the chromogranins and secretogranins pass through the ER and Golgi before entering the granules. Hence, the CGB- and SgII-labeling gold particles were also found in the ER. But the granin proteins are known to be absent in mitochondria [51], [60].

**Figure 3:**
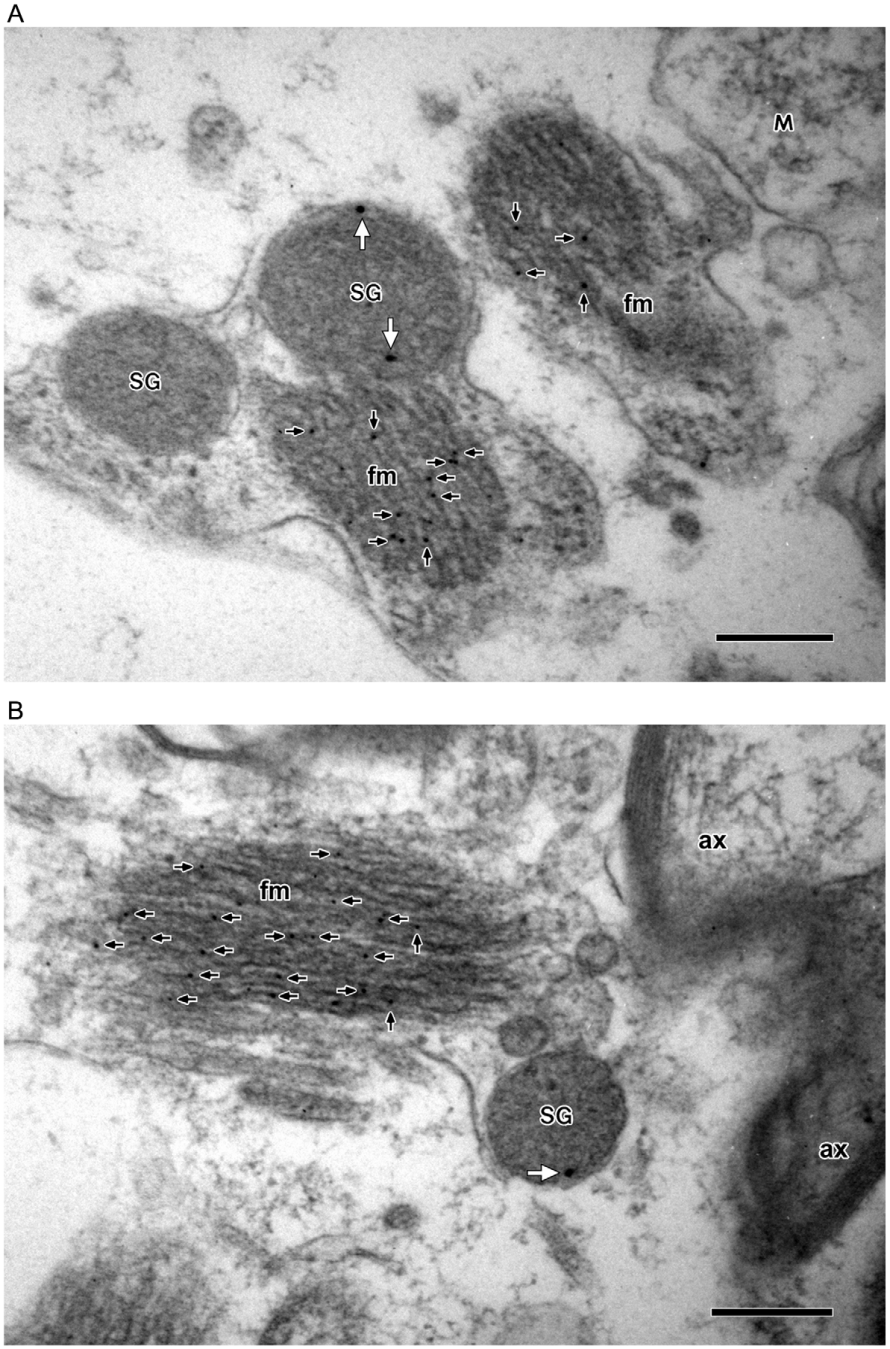
Localization of glial fibrillary acidic protein, CGB, and SgII in astrocytes of brain tissues. Expression of glial fibrillary acidic proteins in the intermediate filaments of astrocytes that contain secretory granules was examined by double immunogold labeling using the antibodies specific for GFAP and either CGB (**A**) or SgII (**B**). (**A**) The GFAP-labeling gold particles (10 nm) and the CGB-labeling gold particles (15 nm) are marked by black and white arrows, respectively. Notice that the GFAP-labeling gold particles are exclusively localized in the filaments (fm) whereas the CGB-labeling particles are limited to secretory granules (SG). (**B**) The GFAP-labeling gold particles (10 nm) and the SgII-labeling gold particles (15 nm) are marked by black and white arrows, respectively. Again the GFAP-labeling gold particles are exclusively localized in the filaments (fm) whereas the SgII-labeling gold particles are localized in secretory granules (SG), but not in mitochondria (M) and axon (ax). Bar = 200 nm.

Furthermore, in light of the presence of the intermediate filaments in the cytoplasm of astrocytes, but not in neurons or other glial cells [56], [57], and of the exclusive expression of glial fibrillary acidic proteins (GFAP) in the intermediate filaments of astrocytes [56]–[59], we have also carried out double immunogold labeling experiments using both the GFAP- and CGB- or SgII-specific antibodies (Fig. 3). As shown in Fig. 3A, the GFAP-labeling gold particles (10 nm) localized exclusively to the intermediate filaments, but not to secretory granules or other structures, while the CGB-labeling gold particles (15 nm) localized to the LDCVs, thereby confirming not only the identity of these cells as astrocytes but also the presence of secretory granules in astrocytes. In addition, the presence of GFAP-labeling intermediate filaments has also been demonstrated along with the SgII-labeling secretory granules (Fig. 3B), thus further identifying the secretory granule-containing cells as astrocytes.

In clear distinction from small synaptic-like vesicles, these secretory granules are large with diameters of 300–400 nm, though it is not uncommon to see larger granules with diameters of >400 nm. Nevertheless, the size is generally comparable to secretory granules of typical neuroendocrine chromaffin cells of human and bovine [60], but is markedly bigger than those of rat or mouse. Moreover, as it is often the case with secretory granules of other secretory cells some secretory granules appear to lack the electron dense intragranular contents, thus looking more transparent in some regions of the granules than others (cf, Fig. 2A). These granules with partly transparent inside may represent vesicles that are either endocytosed (recycled) recently or in the process of maturation (loading).

The relative distribution of the CGB- or SgII-labeling gold particles in secretory granules and mitochondria of human astrocytes is summarized in Table 1. As shown in Table 1, the number of CGB-labeling gold particles per µm^2^ of secretory granule area in astrocytes was 12.38 while that per µm^2^ of mitochondria was 0.37, a background number, thus clearly demonstrating the presence of CGB in secretory granules. Similar to CGB, the number of SgII-labeling gold particles per µm^2^ of secretory granule area in astrocytes was 10.31 while that per µm^2^ of mitochondria was 0.20 (Table 1), a background number, again clearly indicating the presence of SgII in secretory granules. Our approximate estimation of the number of the CGB- and SgII-labeling gold particles per unit area of secretory granules and of the ER appeared to suggest relatively higher concentrations of CGB and SgII in secretory granules than in the ER of astrocytes, as was the case in chromaffin cells [60].

**Figure 4:**
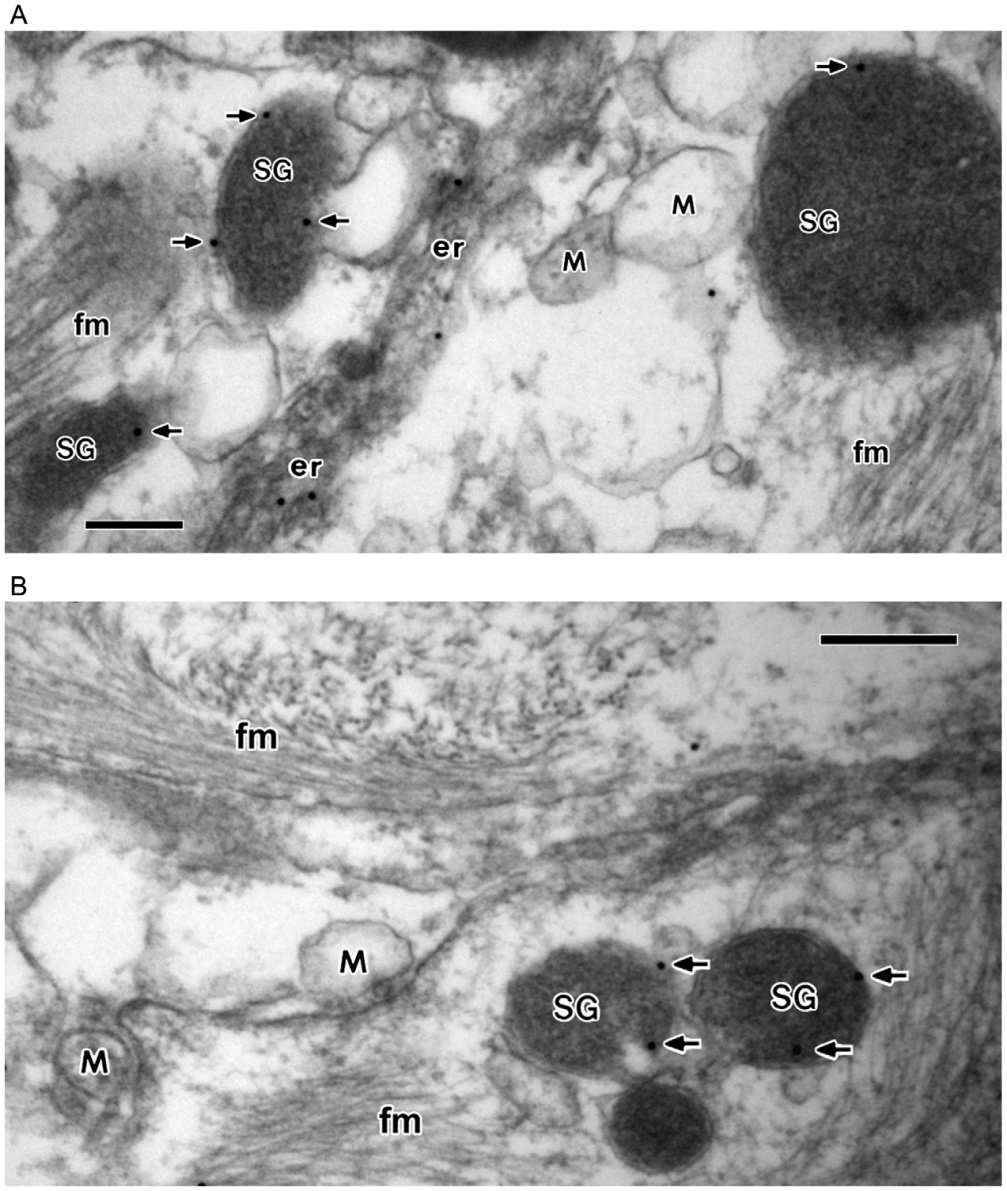
Immunogold electron microscopy showing the localization of IP_3_R1 in secretory granules in astrocytes. Astrocytes from human brain tissues were immunolabeled for IP_3_R1 (15 nm gold) with the affinity purified IP_3_R1 antibody (**A** and **B**). The IP_3_R1-labeling gold particles (indicated by arrows) were primarily localized in the membranes of secretory granules (SG) with some in the endoplasmic reticulum (see **A**), but not in the mitochondria (M). In the control experiments without the primary antibody no gold particles were seen in secretory granules (not shown). fm, filament. Bar = 200 nm.

Moreover, in view of the presence of the IP_3_R/Ca^2+^ channels in secretory granules [52], [55], [61], [62], and of secretory granules serving as the major IP_3_-sensitive intracellular Ca^2+^ stores in secretory cells, the possibility of secretory granules of astrocytes functioning as an IP_3_-sensitive intracellular Ca^2+^ store of astrocytes also arose. Therefore, to investigate the possibility of astrocyte secretory granules serving as an IP_3_-sensitive intracellular Ca^2+^ store, we examined the potential expression of the IP_3_Rs in secretory granules of astrocytes (Figures 4–6). Given the presence of all three isoforms of IP_3_Rs in secretory granules of secretory cells we examined the presence of three isoforms of IP_3_Rs in the astrocyte secretory granules by immunogold electron microscopy using the IP_3_R1-, IP_3_R2-, and IP_3_R3-specific antibodies (Figures 4–6).

**Figure 5 pone-0011973-g005:**
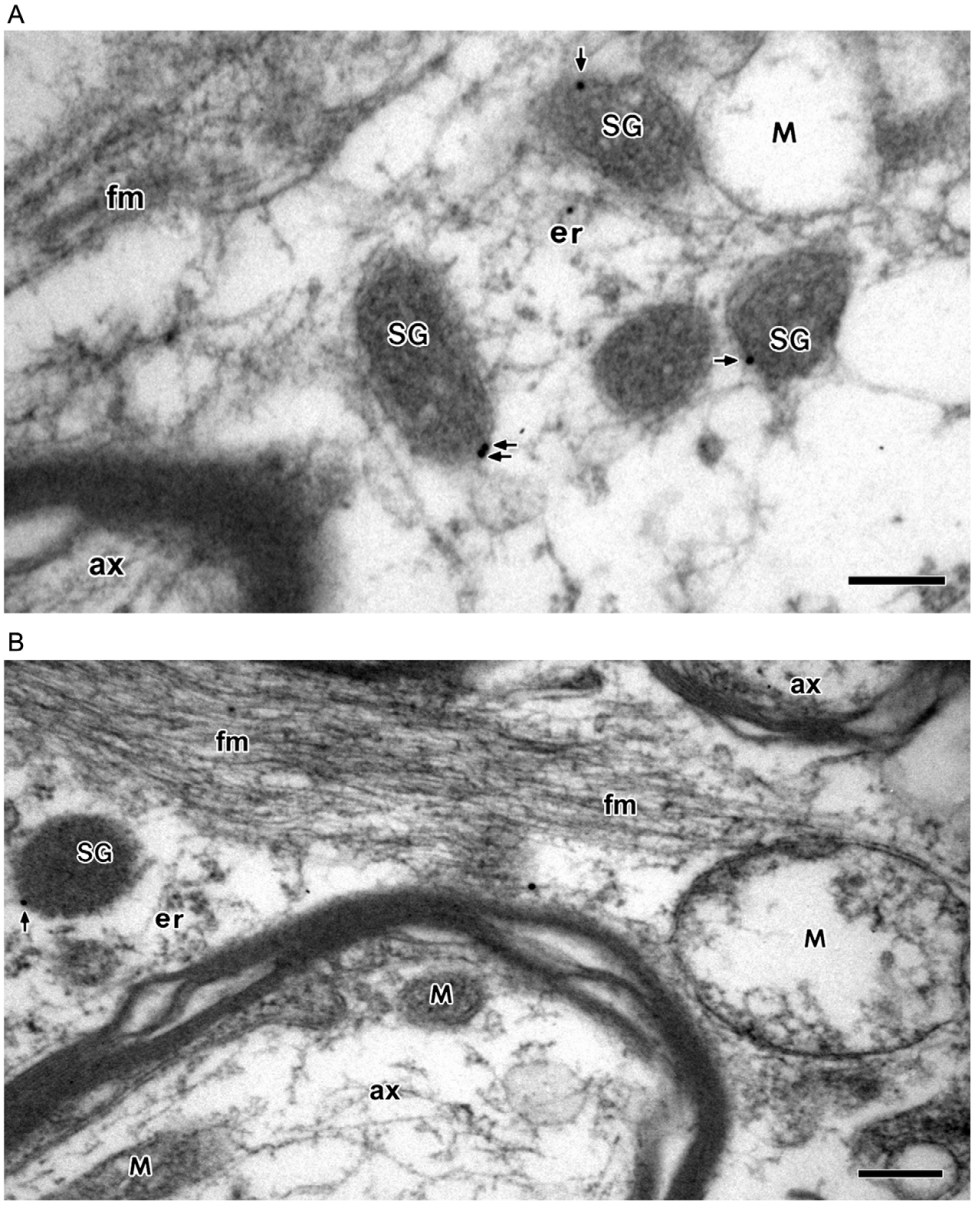
Immunogold electron microscopy showing the localization of IP_3_R2 in secretory granules in astrocytes. Astrocytes from human brain tissues were immunolabeled for IP_3_R2 (15 nm gold) with the affinity purified IP_3_R2 antibody (**A** and **B**). The IP_3_R2-labeling gold particles (indicated by arrows) were primarily localized in the membranes of secretory granules (SG) with some in the endoplasmic reticulum (see **A**), but not in the mitochondria (M). In the control experiments without the primary antibody no gold particles were seen in secretory granules (not shown). ax, axon; fm, filament. Bar = 200 nm.

**Figure 6:**
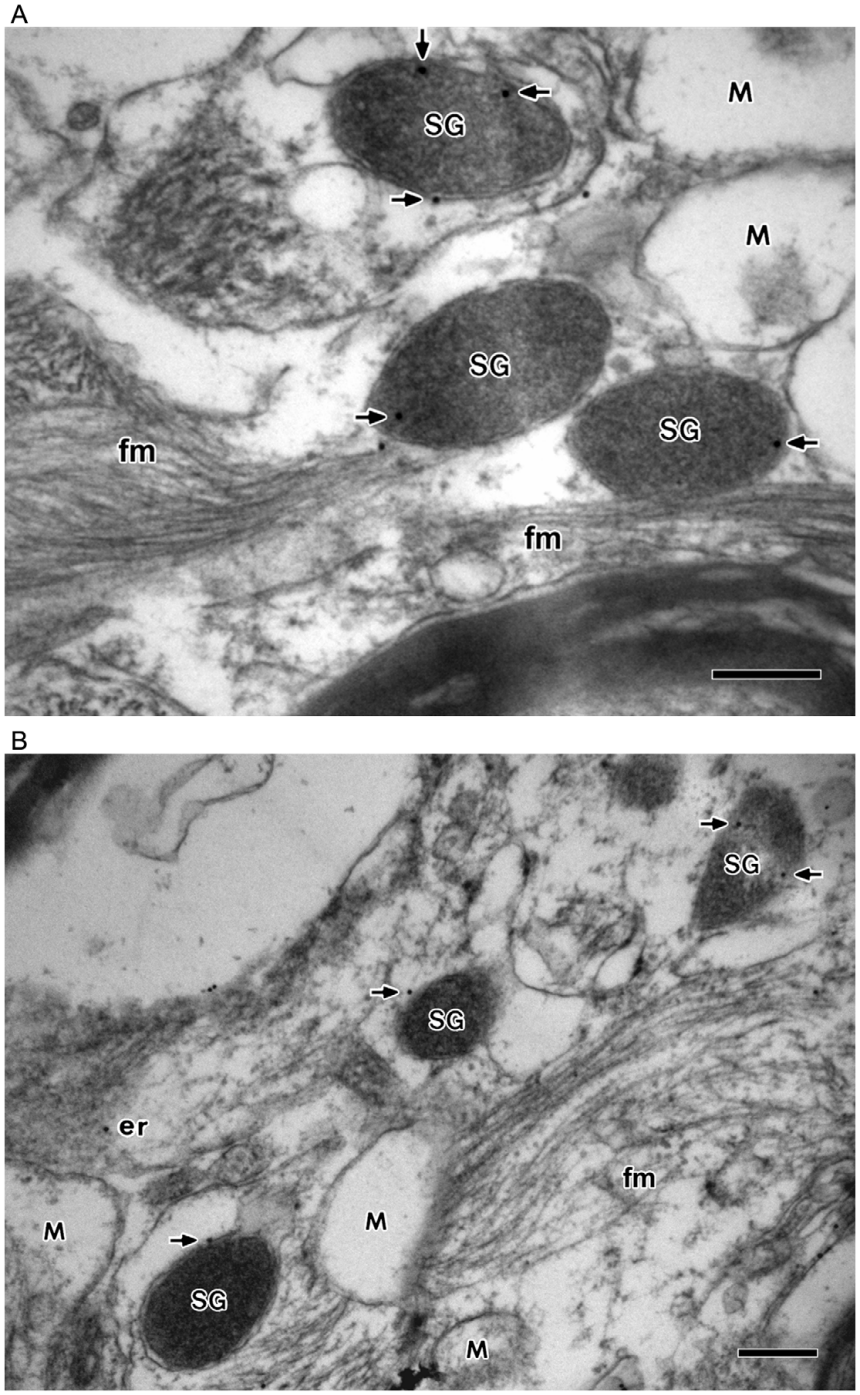
Immunogold electron microscopy showing the localization of IP_3_R3 in secretory granules in astrocytes. Astrocytes from human brain tissues were immunolabeled for IP_3_R3 (15 nm gold) with the affinity purified IP_3_R3 antibody (**A** and **B**). The IP_3_R3-labeling gold particles (indicated by arrows) were primarily localized in the membranes of secretory granules (SG) with some in the endoplasmic reticulum (see **B**), but not in the mitochondria (M). In the control experiments without the primary antibody no gold particles were seen in secretory granules (not shown). ax, axon; fm, filament. Bar = 200 nm.

Consistent with the presence of the IP_3_Rs in secretory granules of other secretory cells [52], [55], [61], [62] and following the nature of the IP_3_Rs being the membrane protein [63], the IP_3_R1-labeling gold particles were localized in the membranes of secretory granules of astrocytes (Fig. 4, A and B). Keeping with the known absence of the IP_3_Rs in mitochondria there were no IP_3_R1-labeling gold particles in mitochondria. Further, astrocyte secretory granules were also shown to localize the type 2 IP_3_R (IP_3_R2)-labeling gold particles (Fig. 5, A and B) and the type 3 IP_3_R (IP_3_R3)-labeling gold particles (Fig. 6, A and B). As was the case in the IP_3_R1, the IP_3_R2- and the IP_3_R3-labeling gold particles were localized primarily along the membranes of secretory granules, but were absent in mitochondria.

The IP_3_R1-, IP_3_R2- and IP_3_R3-labeling results are summarized in Table 2. The number of IP_3_R1-labeling gold particles per µm of secretory granule membrane was 0.872 while that of mitochondria was 0.035, a value considered to be background, clearly demonstrating the presence of IP_3_R1 in secretory granule membranes of astrocytes, but not in mitochondrial membranes (Table 2). Further, the number of IP_3_R2-labeling gold particles per µm of secretory granule membrane was 0.586 while that of mitochondria was 0.022, a value close to virtual zero, which again demonstrated the presence of IP_3_R2 in secretory granule membranes of astrocytes, but not in mitochondrial membranes (Table 2). Similar to the results shown for IP_3_R1 and IP_3_R2, the number of IP_3_R3-labeling gold particles per µm of secretory granule membrane was 0.719 while that of mitochondria was 0.018, a value considered to be background. This result also showed the localization of IP_3_R3 in secretory granule membranes, but not in mitochondrial membranes (Table 2). Interestingly, these results that confirmed the presence of all three isoforms of IP_3_Rs in secretory granules of astrocytes are in complete agreement with the results obtained with secretory granules of typical neuroendocrine chromaffin cells [39], [52], [60].

In line with the previous results on secretory granules that showed the presence of Ca^2+^ storage proteins chromogranins A and B, and secretogranin II, and the IP_3_R/Ca^2+^ channels [41], [52], [55], [62], [64], the above results show that astrocyte secretory granules are also equipped with the necessary machinery that is required to function as a major IP_3_-sensitive intracellular Ca^2+^ store.

## Discussion

Although astrocytes are not traditionally regarded as secretory cells, it is nonetheless evident that they store a variety of molecules that are secreted in a regulated manner and participate in the signaling pathways in the brain. Hence, in spite of the dearth of information regarding exocytosis in glial cells compared to neurons, exocytotic activity in astrocytes is deemed essential in the astrocyte-to-neuron communication that is increasingly considered important for normal function of the brain. Astrocytes are known to contain many gliotransmitters such as glutamate, ATP, D-serine, and regulatory peptides neuropeptide Y (NPY) and atrial natriuretic peptide (ANP) [4], [10], [14], [19]–[28], [30], and these are secreted in a Ca^2+^-dependent regulated exocytotic pathway. The regulated exocytosis in all secretory cells is generally controlled by the cytoplasmic Ca^2+^ concentrations ([Ca^2+^]c), and a sudden increase of cytoplasmic Ca^2+^ concentration is the trigger signal for exocytotic processes.

Large molecules such as regulatory peptides NPY and ANP are primarily stored in the LDCVs [9], [10], [19], [28], [30] while small molecules are stored in the small synaptic-like vesicles [4], [14], [20]–[27] although some such as ATP and glutamate are found in both types of vesicles [10], [27]–[30]. Glutamate has been thought to be released from small synaptic-like vesicles of astrocytes. Yet in recent studies glutamate is also shown to be released from large dense core vesicles with a diameter of ∼310 nm [28], [29] in a Ca^2+^- and SNARE protein-dependent manner [5], [13], [16], [28]. Another prominent signal molecule ATP is also released from the LDCVs in a Ca^2+^ dependent manner [5], [10], [27]. Of particular interest is that secretogranin II, a protein with ∼590 amino acids [65]–[68], is among the large peptides and proteins that are known to exist and released in astrocytes in response to increased [Ca^2+^]c [69]–[71].

Chromogranins and secretogranins are marker proteins of secretory granules [43], [46]–[48] that are a signature organelle for secretory cells. Of these, chromogranins A and B and secretogranin II are three major members of the granin family proteins. Hence the existence of secretogranin II in astrocytes [69]–[71] has implied the presence of secretory granules in astrocytes. As shown in Fig. 2, chromogranin B and secretogranin II are exclusively localized in the large dense core vesicles, thereby identifying the LDCVs as bona fide secretory granules. Chromogranins A and B and secretogranin II are high-capacity, low-affinity Ca^2+^ storage proteins, binding 30–93 mol of Ca^2+^/mol of protein with dissociation constants (Kd) of 1.5–4.0 mM [52], [56], [72], thus enabling secretory granules to store up to ∼40 mM Ca^2+^ [35], [36], the highest concentrations of Ca^2+^ in any subcellular organelles. These proteins are also released, along with other secretory granule contents, in response to stimuli that elevate [Ca^2+^]c.

The elevation in the cytoplasmic Ca^2+^ concentrations in astrocytes is thought to depend on Ca^2+^ release from intracellular Ca^2+^ stores [19], [20], [22], [25], [31]. Likewise, NPY release from the LDCVs is also closely linked to the release of Ca^2+^ from intracellular stores [19]. Of particular interest is the observation that the phospholipase C/inositol phosphate pathway is linked to the release of Ca^2+^ from internal Ca^2+^ stores of astrocytes [20], [22], [25], [31], thereby specifically implicating IP_3_-dependent intracellular Ca^2+^ stores in the Ca^2+^-dependent secretory pathway of these cells. Moreover, in light of the fact that the IP_3_-senstive intracellular Ca^2+^ stores provide sufficient amounts of Ca^2+^ to initiate the secretory processes even in the absence of external calcium [41], [73], [74], it is imperative to identify the intracellular Ca^2+^ stores to understand not only the intracellular Ca^2+^ control mechanisms but also the regulated secretory pathway of astrocytes.

Secretory granules of bovine chromaffin cells contain the highest concentrations of all three isoforms of IP_3_R3, containing 58–69% of total cellular IP_3_Rs [39]. In addition, chromogranins A and B bind directly to the IP_3_Rs at the intragranular pH 5.5 [75], [76] and activate the IP_3_R/Ca^2+^ channels, i.e., increase both the mean open time and the open probability of the channels upon IP_3_ binding, 9–42-fold and 8–16-fold, respectively [77]–[79]. Therefore, given that secretory granules contain the majority of cellular chromogranins A and B and of all three isoforms of IP_3_Rs, and that the coupling between the chromogranins and the IP_3_Rs changes the structure of the IP_3_R/Ca^2+^ channels to a more ordered and open-ready state [79] it appears natural for secretory granules to function as the major IP_3_-sensitive intracellular Ca^2+^ store of the cells in which they are localized.

**Figure 7:**
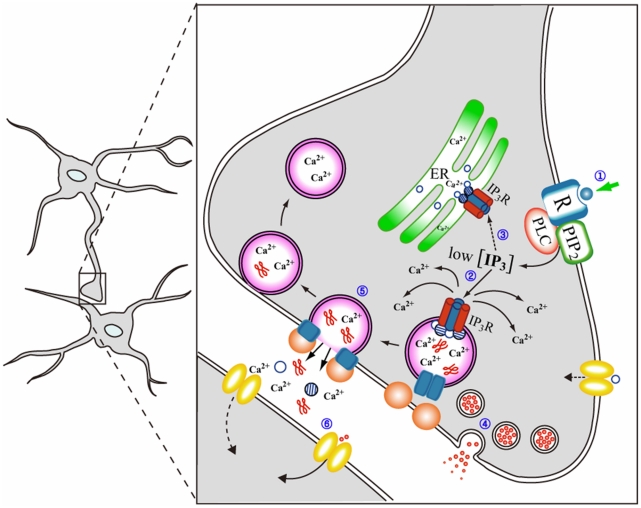
A model showing the IP_3_-induced Ca^2+^ mobilization from secretory granules and the secretory processes of astrocytes. The tetrameric IP_3_R/Ca^2+^ channels are shown in red and blue columns while chromogranins A and B are shown in open and hatched circles, respectively. Only can chromogranin B, which interacts with CGA to form a CGA-CGB heterodimer at the pH of ER, couple to the tetrameric IP_3_Rs in the ER [75] whereas both chromogranins A and B, which form a CGA_2_CGB_2_ heterotetrameric complex at the acidic intragranular pH [94], couple to the tetrameric IP_3_Rs in secretory granules [75], [77], [78]. Stimuli at the cell surface (1) will lead to the production of IP_3_ at the plasma membrane, which will serve as the first signal to induce the IP_3_-dependent Ca^2+^ release from intracellular Ca^2+^ stores in the cytoplasm. Yet each intracellular Ca^2+^ store will respond differently to IP_3_ depending on the amount of IP_3_ produced and the sensitivity of the IP_3_R/Ca^2+^ channels to IP_3_. In light of the significantly higher sensitivity of the IP_3_R/Ca^2+^ channels of secretory granules than those of the ER [40], secretory granules will release Ca^2+^ (2), ahead of the ER (3), in response to low IP_3_ concentrations. This Ca^2+^ could play essential roles in initiating secretion by synaptic-like vesicles (4) and secretory granules (5), leading to secretion of the gliotransmitters (6).

However, unlike the acidic intragranular milieu of secretory granules [80], [81] the pH of the ER is maintained ∼7.4 [82]–[84], and at this physiological pH, chromogranin A fails to bind the IP_3_Rs directly and only chromogranin B remains bound to the IP_3_Rs [75] (Fig. 7). Yet the binding strength of CGB to the IP_3_Rs at a near physiological pH 7.5 is significantly weaker than that at the intragranular pH 5.5 [75], [85], and as a result the IP_3_R/Ca^2+^ channel-activating effect of CGB at this pH is markedly weaker than that shown at pH 5.5 [77], [78]. As though to reflect accurately the differences in the physiological conditions of secretory granules and the ER, the secretory granule IP_3_R/Ca^2+^ channels are shown to be at least 6–7-fold more sensitive to IP_3_ than those of the ER [40], which means that secretory granules will be able to release Ca^2+^ in response to an IP_3_ concentration that is lower than one-seventh that is required to induce IP_3_-dependent Ca^2+^ release from the ER. In other words, the markedly higher IP_3_ sensitivity of the secretory granule IP_3_R/Ca^2+^ channels indicates that secretory granules will be able to sense the arrival of IP_3_ long before the ER can and respond by releasing the granular Ca^2+^ ahead of the ER (Fig. 7). It is highly likely that this Ca^2+^ would play key roles in initiating the exocytotic processes by the secretory granules and to a certain extent by synaptic-like vesicles as well, resulting in the secretion of ions and gliotransmitters that participate in the cell-to-cell communication. Moreover, in light of the fact that the SNARE protein syntaxin 1A and synaptotagmin I have been shown to exist in secretory granules of chromaffin cells and interact with chromogranins A and B [86] and that cellubrevin (VAMP3), synaptobrevin 2 and synaptotagmin were shown to colocalize with secretory granule markers in anterior pituitary cells [87], it is highly likely that SNARE proteins also exist in secretory granules of astrocytes.

Considering that secretory granules are present in all types of secretory cells (neurons, endo/exocrine cells, and neuroendocrine cells), the presence of secretory granules in astrocytes is in line with the already established secretory activity of these cells in the brain. In particular, the rich presence of chromogranin B and secretogranin II and the three IP_3_R isoforms in secretory granules of astrocytes is in full agreement with the distribution of these molecules in secretory granules of typical neuroendocrine chromaffin cells [39], [60], which function as the major IP_3_-sensitive intracellular Ca^2+^ stores. Indeed, our preliminary studies show that IP_3_ mediates Ca^2+^ release in cultured astrocytes even in the condition in which the ER Ca^2+^ is depleted due to the presence of thapsigargin (Yoo et al., unpublished results), whereby strongly suggesting the IP_3_-dependent Ca^2+^ release from secretory granules of astrocytes. Further, in view of the observation that the cell processes of astrocytes appear to contain more secretory granules than the cell bodies and that the presence of secretory granules in the cell processes appears to be fairly common, the fine control of IP_3_-dependent Ca^2+^ signaling mechanism in the cell processes will be all the more important in controlling the exocytotic activity of astrocytes through the cell processes, which is vital in cell-to-cell communication in the brain. Yet the situation will be different in cultured astrocytes. That secretory granules of cultured astrocytes appeared to distribute evenly in the cytoplasm [9] may have resulted from the directionless culture conditions.

The major IP_3_-sensitive intracellular Ca^2+^ store role of secretory granules has been demonstrated with many other types of secretory cells, such as chromaffin cells, pancreatic β- and acinar cells, mast cells and airway goblet cells [41], [88]–[92], and the IP_3_-induced intracellular Ca^2+^ release from secretory granules in the absence of external Ca^2+^ has been proven to be sufficient to initiate the exocytotic processes [41], [73], [74]. It was further shown recently that Ca^2+^ release through the IP_3_R/Ca^2+^ channels of secretory granules in pancreatic acinar cells is primarily responsible for the initiation of alcohol-related acute pancreatitis [93]. Therefore, given that the presence of secretory granules in the cell increases not only the magnitude of IP_3_-dependent cytoplasmic Ca^2+^ release but also the IP_3_ sensitivity of the cytoplasmic IP_3_R/Ca^2+^ channels of the cell [34], the presence of secretory granules in astrocytes is expected to contribute to both the large amounts of Ca^2+^ released in the cytoplasm and the high IP_3_ sensitivity of the cytoplasmic IP_3_R/Ca^2+^ channels of astrocytes. In this regard, the recent studies that showed the requirement of IP_3_-mediated intracellular Ca^2+^ releases for the increased expression and secretion of fibroblast growth factor-2, which has a size of ∼24 kDa, by astrocytes [31] and the critical role of Ca^2+^ release through the IP_3_R/Ca^2+^ channels for the proliferation, motility, and invasion of human astrocyte cancer cells [42] appear to underscore the importance of the IP_3_-dependent Ca^2+^ signaling in the physiology of astrocytes.
